# Emerging Roles of Extracellular Vesicles in the Central Nervous System: Physiology, Pathology, and Therapeutic Perspectives

**DOI:** 10.3389/fncel.2021.626043

**Published:** 2021-02-23

**Authors:** Yadaly Gassama, Alexandre Favereaux

**Affiliations:** Univ. Bordeaux, CNRS, Interdisciplinary Institute for Neuroscience, IINS, UMR 5297, Bordeaux, France

**Keywords:** extracellular vesicles, exosomes, central nervous system, brain, neurodegenerative diseases, glioma, therapies, biomarkers

## Abstract

Extracellular vesicles or EVs are secreted by most, if not all, eukaryote cell types and recaptured by neighboring or distant cells. Their cargo, composed of a vast diversity of proteins, lipids, and nucleic acids, supports the EVs’ inter-cellular communication. The role of EVs in many cellular processes is now well documented both in physiological and pathological conditions. In this review, we focus on the role of EVs in the central nervous system (CNS) in physiological as well as pathological conditions such as neurodegenerative diseases or brain cancers. We also discuss the future of EVs in clinical research, in particular, their value as biomarkers as well as innovative therapeutic agents. While an increasing number of studies reveal EV research as a promising field, progress in the standardization of protocols and innovation in analysis as well as in research tools is needed to make a breakthrough in our understanding of their impact in the pathophysiology of the brain.

## Introduction

While neurotransmitters or chemokines are released by specific cell types, extracellular vesicles (EVs) are a way for most, if not every, cell types to transfer information to their surroundings or even distant targets, as these vesicles are found in different fluids such as the cerebrospinal fluid (CSF), blood, or urine (Meng et al., 2019).

Long thought to be a way for the cell to get rid of unnecessary compounds, EVs are now considered an integral part of cell–cell communication.

Although the EVs’ classification is still a matter of debate, EVs can be separated into two categories depending on their biogenesis: (i) microvesicles (size ranging from 100 to 1,000 nm) that come from the budding of the cell membrane (Tricarico et al., 2016) and (ii) exosomes (size ranging from 30 to 150 nm), which are derived from the endocytic pathway. The multivesicular body (MVB) originates from the budding of the late endosome-forming intraluminal vesicles (ILVs); then, it either fuses with a lysosome, causing the degradation of the MVB content, or the MVB can fuse to the plasma membrane, releasing ILVs (exosomes) in the extracellular space. Once in the extracellular space, exosomes can deliver their content to virtually any cell type and affect the physiology of the receiving cell (van Niel et al., 2018; Mashouri et al., 2019). This aspect of EV biogenesis has been thoroughly described by van Niel, D’Angelo, and Raposo in a recent review (van Niel et al., 2018). Indeed EVs can contain several effectors such as transmembrane (e.g., tetraspanins) or soluble proteins (e.g., syntenin-1 or heat shock proteins) and lipids that can be involved in cell signaling (Gabrielli et al., 2015). Nucleic acids are also part of EVs’ cargo such as miRNAs which are short non-coding RNAs able to target mRNAs and silence translation (Skog et al., 2008). Messenger RNAs are also commonly found inside EVs, but their impact on a recipient cell is still a debate, notably due to the restricted half-life of mRNAs and also because the mRNAs found in EVs may well be fragmented. The cargo molecules transported by EVs are intensively explored and the subject of numerous specialized databases like Vesiclepedia or EVpedia (Kalra et al., 2012; Kim et al., 2013).

In this mini-review, we highlight a selection of works to show the implication of EVs in the physiological mechanisms of the central nervous system (CNS), then we will describe the roles of EVs in CNS disorders such as neurodegenerative diseases, and finally we will discuss the potential of EVs as an early diagnostic tool and their impact in the development of new therapeutic approaches.

## Physiological Role of EVs in the CNS

First discovered in immune cells (Raposo et al., 1996), EVs were later discovered in the CNS where neurons (Fauré et al., 2006; Morel et al., 2013), astrocytes (Tytell, 2005), microglia (Potolicchio et al., 2005), and oligodendrocytes (Krämer-Albers et al., 2007) were shown to release EVs. However, our understanding of the physiological role of EVs released by CNS cells is still partial, mainly due to the lack of technology enabling the study of EVs produced by specific cell types *in vivo*. Another limitation is the intrinsic physiology of neurons: the release of synaptic vesicles and the release of EVs have some overlapping cellular mechanisms. Thus, studying EV release by loss-of-function approaches would cause a dramatic impairment to the physiology of the neuron that could not be attributed solely to the inhibition of EVs’ release. Yet some discoveries have been made using cell culture models (collected in Table 1) highlighting the potential role of EVs in the physiology of the synapse as well as the potential implication in CNS disorders.

**TABLE 1:** Main features of CNS EVs: physiological properties, pathological implications, biomarkers and therapeutic uses.

**Physiological conditions**			
**Cell type**	**Paradigm**	**Cargo**	**References**
Neurons	Neurons’ EVs involved in synaptic transmission and retrograde signaling	GluR2/3, Stg4	Fauré et al., 2006 and Korkut et al., 2013
	Neurons’ EVs linked to neurodevelopmental processes	miR-132	Xu et al., 2017
Astrocytes	Neurite development	Synapsin-I	Wang et al., 2011
	Neuroprotection from oxidative stress	ApoD	Pascua-Maestro et al., 2019
Microglia	Oligodendrocyte differentiation	PTPRZ	Willis et al., 2020
Oligodendrocytes	Inflammation-related content	miR-146a, IL-1ß	Prada et al., 2018; Lemaire et al., 2019, and Bianco et al., 2005
	Neuroprotection from oxidative stress	–	Fröhlich et al., 2014
**Pathological conditions**			
**Disease**	**Mechanisms**	**Cargo**	**References**
Alzheimer’s disease (AD)	Development and spreading of AD lesions	Aß peptide, tau/miR-193b	Vingtdeux et al., 2012; Delpech et al., 2019, and Liu et al., 2014
Parkinson’s disease	Aggregate formation and dissemination	a-Synuclein	Stuendl et al., 2016 and Emmanouilidou et al., 2010
Huntington disease	Toxicity leading to neurodegeneration in Huntington disease	Extended RNA repeats and polyQ proteins	Zhang et al., 2016
Glioma	Decreased c-myc levels and activation of glial cells	miR-451, miR-21	D’Asti et al., 2012 and Oushy et al., 2018
	Transfer of cancer-like phenotypes	EGFRvIII	Al-Nedawi et al., 2008
	Temozolomide resistance	miR-1238	Yin et al., 2019
Multiple sclerosis	Inflammation	Il-1ß	Verderio et al., 2012 and Sbai et al., 2010
	Degradation of the extracellular matrix	MMP, caspase-1	
**Biomarkers**			
**Disease**	**Biofluid**	**Cargo**	**References**
Glioblastoma	Serum	EGFRvIII mRNA	Lemaire et al., 2019
Alzheimer’s disease	CSF	tau, Aβ42, and IRS-1	Fiandaca et al., 2015; Goetzl et al., 2015, and Kapogiannis et al., 2019
**Therapies**			
**Disease**	**Target**	**Cargo/Modification**	**References**
Neuroinflammation	All central nervous system cells	Curcumin	Devassy et al., 2015; Unlu et al., 2016, and Wang et al., 2016
Neurodegenerative diseases	Neurons only	Lamp2b modified with RVG motif	Alvarez-Erviti et al., 2011

### Neurons

Inter-cellular communication in the CNS is a crucial mechanism for higher brain functions. Neurons are highly specialized cells able to transfer neurotransmitters through chemical synapses and ions through gap junctions (electrical synapses) or release neuropeptides via dense core vesicle release. In addition, neurons have also been shown to release EVs in cell culture models (Fauré et al., 2006). Morel et al. (2013) showed that neurons release EVs, while Sadoul’s team linked the neuronal EVs’ release to their synaptic activity (Lachenal et al., 2011). Indeed a prolonged (3 h) incubation, with a high extracellular KCl concentration causing a strong depolarization of neurons, induced an increase of EV release in both developing and mature neurons (Chivet et al., 2014). It has been hypothesized that EVs could be involved in neuronal excitation in a Ca^2+^-like manner (Lachenal et al., 2011). Neurotransmitter receptors such AMPAR subunits GluR2/3 are reported to be present in these neuron derived-EVs, suggesting their role in post-synaptic plasticity by potentially modifying the recipient cell’s excitability.

Extracellular vesicles derived from neurons also seem to be implicated in retrograde signaling using the transfer of Synaptotagmin4 (Stg4), a post-synaptic calcium sensor. Stg4 activation induces a signaling cascade involving cAMP, leading to a pre-synaptic stimulation (Korkut et al., 2013). Here they show that the required transfer of Stg4 from the pre- to the post-synaptic neuron, enabling the retrograde control to happen, is at least partially mediated by EVs.

The release of EVs is still a hot topic, as some studies showed potential vesicle secretion from the neurites (Goldie et al., 2014) that could be complementary to the expected release of EVs from the soma of the neuron. Indeed they showed a specific depletion of miRNAs in neurites following depolarization and an enrichment of the same miRNAs in vesicles collected from the depolarization media. In addition to the protein content, the nucleic acid content of neuron-derived EVs (nEVs) seems to be involved in multiple processes in the CNS-like neurological development. For instance, miR-132, a common neuronal EV miRNA targeting Ctbp2 (C-terminal binding protein 2), which is involved in regulating Notch signaling cascade, leads to an accumulation of glial progenitor cells (Xu et al., 2017).

### Astrocytes

In essence, astrocytes are a cell type providing brain homeostasis as well as neurodevelopmental support through multiple processes. Astrocyte EV cargo contains synapsin-I, suggesting an involvement of these EVs in neurite development (Wang et al., 2011). Interestingly, under specific conditions, astrocyte-derived EVs contain proteins involved in promoting neuronal survival, neuroprotection (Martínez-Pinilla et al., 2015; Pascua-Maestro et al., 2019), or synaptic transmission (Chaudhuri et al., 2020). Astrocytes are also known for their role in neuroprotection, notably against oxidative stress where EVs are also involved. For instance, apolipoprotein D (ApoD), a glycoprotein implicated in the maintenance of neuronal function and integrity under oxidative stress conditions, is transported via EVs (Martínez-Pinilla et al., 2015). Indeed Pascua-Maestro and colleagues showed an increase in neuron survival when exposed to astrocytic cell-conditioned media compared to ApoD-KO astrocytic cell-conditioned media. They also showed the presence of ApoD monomer and dimers in EVs, suggesting that the neuroprotective role and transfer of ApoD are mediated by EVs, at least partially, pending the confirmation that astrocytic EVs do have the same effect as astrocytic-conditioned media (Pascua-Maestro et al., 2019).

Moreover, aside from their implication in neuronal development, astrocyte-derived EVs affect oligodendrocytes: PTPRZ, a tyrosine phosphatase involved in the differentiation of oligodendrocyte progenitor cells into oligodendrocytes, has been found in young astrocytic EVs, strongly suggesting a role of EVs in this maturation (Willis et al., 2020).

### Other Glial Cells

Microglia are the CNS’s resident macrophages and are greatly responsible of the active immunity in this area. As immune cells, they are able to release cytokines, chemokines, and other soluble factors that are linked to immune responses. Similarly to astrocytes, microglia derived-EVs are also involved in multiple processes like neuroprotection. Hence, Prada et al. (2018) found miR-146a, a miRNA associated to several inflammation-related diseases, to be enriched in microglia-derived EVs. This finding was later confirmed by a study of Lemaire et al. (2019). As CNS sentinels, microglia are also responsible for the overall homeostasis of the brain: they are able to sense extracellular ATP, which may be the result of damaged cells, and to react to these high ATP levels by shedding EVs containing pro-inflammatory factors, like IL-1β, thus enhancing neuroinflammation (Bianco et al., 2005).

Oligodendrocytes are differentiated cells mainly known for the generation of myelin sheath, a lipid-composed membrane that is enabling axonal insulation and increased-velocity action potentials. Regarding oligodendrocyte-derived EVs, not much information is available, but multiple studies by the Krämer-Albers group (Frühbeis et al., 2013; Fröhlich et al., 2014) showed a neuron–oligodendrocyte crosstalk mediated by EVs: the secretion of EVs by oligodendrocytes ensured an increased viability of the neurons under stress conditions (such as oxidative stress). Indeed they showed increased metabolic activity in neurons growing under stress conditions (nutrient deprivation or oxidative stress) in the presence of oligodendrocyte-derived EVs. While the actual mechanisms remain to be elucidated, this study highlights the potential supportive role of these EVs in the CNS.

## Pathological Implication of EVs in CNS Disorders

### Neurodegenerative Diseases

Among CNS disorders, neurodegenerative diseases have received a lot of attention throughout the years, enabling significant progress in understanding pathological mechanisms. Interest toward the potential implication of EVs in these diseases grew, notably due to the ability of EVs to carry proteins and especially pathologic seed proteins such as Aβ, α-synuclein, or tau protein (see Table 1 for details). The theory that EVs are able to propagate these diseases in the brain is still under study, as multiple reports showed that, *in vitro*, EVs from different neural cell types (neurons, astrocytes, oligodendrocytes, microglia) were able to carry these seeding proteins (Vingtdeux et al., 2012; Stuendl et al., 2016; Delpech et al., 2019). Nevertheless, *in vivo* studies are still lacking to prove the propagation potential of EVs.

Proteins are not the only bioactive content of EVs that is studied in these disorders, as miRNAs, through their epigenetic control of recipient cells, proved to have a great impact on the pathological mechanisms of numerous diseases. Studies on CSF from Alzheimer’s disease patients showed a downregulation of miR-193b in EVs (Liu et al., 2014). Interestingly, this miRNA is regulating the mRNA coding for the APP protein, probably leading to increased mRNA translation and inducing an accumulation of the APP protein.

The pathophysiology of Parkinson’s disease (PD) is of great interest, and the role of aggregated α-synuclein protein in neurodegeneration has been well established as a cause for the symptoms associated with PD (bradykinesia, tremors, or dementia). In the recent years, EVs gained interest as a potential actor in PD, and many teams started to focus on their role in the pathophysiology of the disease. As early as 2010, *in vitro* studies showed the α-syn-carrying potential of EVs (Emmanouilidou et al., 2010), paving the way for the extracellular seeding theory. Stuendl et al. (2016) showed that CSF-derived EVs from PD patients are able to cause α-synuclein aggregates in target cells and could confer disease pathology.

The impact of EVs on the pathophysiology of Huntington disease is less studied since it is considered as a rare disease, and the mechanisms involved have yet to be fully elucidated. However, a few laboratories, such as Breakefield and colleagues, decided to test the EV hypothesis in Huntington disease. Using a cell culture model overexpressing the HTT polyglutamine exon, they showed a possible transport of both extended RNA repeat as well as the poly-Q proteins by EVs, opening the door for a potential role of EVs in disease spreading (Zhang et al., 2016).

Finally, multiple sclerosis (MS) is an inflammatory neurodegenerative disease where the neuronal myelin sheets are damaged, leading to various symptoms depending on the amount of nerve damage and which nerves are affected. This neuroimmune disease exists in various forms that are associated with different clinical profiles. EVs’ involvement in inflammation mechanisms has soon been identified as a potential neuroinflammation carrier since they have been shown to carry pro-inflammatory cytokines such as IL-1ß (Bianco et al., 2005). Indeed Verderio et al. (2012) showed that myeloid microvesicles can spread inflammatory signals in the brain of mice and that inhibition of microvesicles releases protected mice from experimental autoimmune encephalomyelitis. Another characteristic of MS is the migration of immune cells in the CNS due to the disruption of the blood–brain barrier, leading to increased inflammation. EVs also seem to be implicated in this pathophysiological process since metalloproteinases (MMP) are commonly found in EVs coming from the endothelial cells of the blood–brain barrier. Indeed these enzymes are involved in extracellular matrix degradation, which could play a role in the integrity of the blood–brain barrier (Sbai et al., 2010). Moreover, caspase-1, an enzyme shown to increase MMP activity, is also found in EVs, which could give a favorable context for the degradation of the extracellular matrix and the intravasation of immune cells in the CNS, leading to inflammation (Sarkar et al., 2009). Even though research is still ongoing, all signs point toward a role of EVs in the propagation of neurodegenerative diseases, and with the development of new tools to study EVs, critical insights into the role of EVs are becoming more accessible.

### Glioma

Due to their ability to modulate other cells, EVs have been extensively studied in the context of tumors and the tumor environment. Gliomas represent a large portion of brain tumors, and among them, glioblastoma multiforme (GBM) is the most aggressive primary brain tumor in adults. Current treatment includes surgery, radiotherapy, and chemotherapy. However, treated patients average a 15-month survival duration after diagnosis due to high recurrence. In order to increase the life expectancy of patients diagnosed with GBM, researchers have focused their attention on the different aspects of this disease and, notably, its ability to communicate with the patient’s immune system. Indeed GBM has been shown to induce the production of cytokines, leading to a pro-tumorigenic environment (D’Asti et al., 2012; Oushy et al., 2018). This observation has been shown for EVs produced from GBM cells generally enriched in miR-451 and miR-21, which leads to decreased levels of c-myc in targeted cells as well as increased activation of glial cells (D’Asti et al., 2012). The production of EVs by GBM cells has also been shown to affect astrocytes, enabling the creation of a pro-tumorigenic environment (Oushy et al., 2018).

Extracellular vesicles are one way for the cancer cells to communicate with non-cancerous cells and have been shown to enable a cross-talk between cancer cells. Indeed oncogenic proteins such as EGFRvIII are also transported from cancer cells to other cancer cells, transferring cancer-like phenotypes such as anchor-independent growth (Al-Nedawi et al., 2008) and leading to the creation of more invasive tumors.

Chemotherapy resistance is a big issue in GBM, and a new mediator of this resistance has been identified in EVs: chemo-resistant cancer cells showed the ability to transfer miR-1238 to other cancer cells via EVs, giving them the ability to resist temozolomide (Yin et al., 2019), one of the most commonly used chemotherapy agent in the treatment of glioblastoma.

## Diagnosis and Therapeutic Perspectives Involving EVs

### Biomarker Discovery

Besides their ability to carry protein through biological barriers, the unique content of EVs produced in specific physiological or pathological conditions is gathering interest. As protein and miRNA content reflects the state of the producing cells, researchers have been looking to identify specific content. EV biomarkers have been quite extensively described in pathologies such as cancer, where EVs can be isolated from serum in order to look for EGFRvIII mRNA to diagnose glioblastoma, bypassing the need for a more invasive tissue biopsy (Skog et al., 2008). In regards to neurodegenerative diseases, some studies showed an abundance of signature molecules inside EVs, and multiple studies have shown potential AD biomarkers (miRNAs and proteins) that could fit with the application of early diagnosis. Indeed Fiandaca et al. (2015) and Goetzl et al. (2015) showed an increase of tau protein as well as lysosomal proteins in AD patients, suggesting that these two protein types could be used as biomarkers. Recently, a clinical study focused on nEVs linked a specific content of nEVs to an Alzheimer’s disease diagnosis (Kapogiannis et al., 2019). Identifying specific thresholds of tau, Aβ42, and IRS-1 as well as nEV concentration from total EV, they were able to establish, with high sensitivity, if a patient from the cohort was indeed an AD patient. This work has established neuron-derived extracellular content as an option to diagnose Alzheimer’s disease (Kapogiannis et al., 2019).

### Therapies

Ever since their discovery, EVs gathered interest for their ability to carry bioactive molecules, hence their potential as therapeutic agents. Due to their ability to circulate in multiple body fluids as well as crossing physiological barriers such as the blood–brain barrier, EVs were soon engineered to carry different compounds such as chemotherapeutic agents, siRNA, or the Cas9 protein. Moreover, depending on the cellular origin, autologous EVs could induce low to no immunogenicity, making them an appropriate tool to deliver different compounds for personalized medicine. In contrast, EVs produced by the dendritic cells from the immune system express major histocompatibility complex class I and II molecules. Interestingly, stimulating dendritic cells with tumor peptides induced the secretion of exosomes with anti-tumor activity (Zitvogel et al., 1998).

Neuroinflammation is linked with several CNS disorders such as MS, granting interest for researchers to find a way to modulate this pathophysiological process with compounds that could not necessarily cross the blood–brain barrier, such as curcumin (Devassy et al., 2015; Unlu et al., 2016; Wang et al., 2016). Curcumin is a phenol compound with a great immunomodulatory effect through the inhibition of cyclooxygenase 2, an enzyme responsible for the production of proinflammatory molecules. It also has an effect on NFκB, a central transcription factor that is linked to the release of different pro-inflammatory cytokines. Zhuang et al. (2011) showed that the delivery of curcumin encapsulated in EVs resulted in the inhibition of lipopolysaccharide-induced brain inflammation. Most of the works on EV-mediated therapy have been focused on the delivery of small RNA to specific, selected cell types. Wood’s team in Oxford has been a pioneer in this area, where they focused on brain delivery of siRNA. In 2011, they showed the ability of exosomes to target specific cells, thanks to a chimeric transmembrane protein, Lamp2b (a protein enriched in endosomal compartment). They modified Lamp2b by adding a virus-derived motif (RVG – derived from the rabies virus) that enables the EVs to be directly targeted to neurons, oligodendrocytes, or microglia (Alvarez-Erviti et al., 2011). This work has opened the door for future therapies to treat neurodegenerative diseases. Indeed, in late 2016, Didiot et al. published a paper where they used EVs loaded with a siRNA directed against huntingtin mRNA, which is responsible for Huntington disease. They showed a dose-dependent decrease of huntingtin mRNA with a 35% decrease in target mRNA expression both *in vitro* and *in vivo* (Didiot et al., 2016).

Extracellular vesicle-based therapies still have a lot of hurdles to overcome as many therapies fail in clinical trials due to physiological limitations diminishing the therapeutic impact. For example, an autologous injection of EVs leads to no immunogenicity; however, the same cannot be said from EVs produced by a cell line or a different person. Moreover, EVs circulating in bodily fluids tend to be cleared by immune cells, resulting in no delivery to the appropriate targets. These therapies may also encounter practical hurdles such as the targeting of EVs to specific organs or cell type (also called tropism). Although many advances have been made, this aspect still needs to be improved, as the therapeutic efficiency is reliant on cell specificity. The efficient loading of cargo inside the vesicles is also a challenge to overcome, but recent papers showed a great improvement in this aspect using hydrophobic RNA molecules or RNA conjugated to cholesterol to increase the loading efficiency (Haraszti et al., 2018).

## Conclusion

The key communication roles of EVs in the physiological mechanisms of the brain seems to be established now. However, the cell-type-specific functions of EVs are still to be determined, but given the striking specification of CNS cells, it is very likely that EVs originating from neurons or astrocytes will not play the same role. Thus, to make a breakthrough in our understanding of EV function in the CNS, we need to design new methodologies to assess, in a cell-specific manner, the content and the communication potential of EVs. Although the impact of EVs in CNS disorders is still an ongoing research, many evidences point toward a crucial physiopathological role of EVs. Finally, the future of EVs certainly lies in biomarker discovery and the development of therapeutic approaches. However, more extensive studies as well as standardized collection methods of EV are needed since isolation methods have a strong influence on EV content due to the level of integrity and purity of the vesicles. To be able to draw conclusions about the biomarker potential of EV, common ground and work must be done to standardize the different protocols used to produce, isolate, and analyze EVs. Efforts toward that direction are increasing, as the EV community continues to propose and implement cohesive guidelines concerning EV preparation and characterization (Théry et al., 2018).

Progress toward EV as therapeutics is being made, including new techniques allowing an increase in the loading of EVs with specific cargoes like miRNA, which could lead to more EV-based therapies going to clinical trials.

## Author Contributions

YG and AF wrote the manuscript. Both authors contributed to the article and approved the submitted version.

## Conflict of Interest

The authors declare that the research was conducted in the absence of any commercial or financial relationships that could be construed as a potential conflict of interest.
